# Complete blood count-based inflammation indexes and symptom severity in people with bipolar disorder: an analysis based on structural equation modelling

**DOI:** 10.1007/s00406-025-02129-2

**Published:** 2025-11-04

**Authors:** Daniele Cavaleri, Cristina Crocamo, Ilaria Riboldi, Pierluca Guzzi, Francesco Bartoli, Giuseppe Carrà

**Affiliations:** School of Medicine and Surgery, University of Milano-Bicocca, via Cadore 48, 20900 Monza, Italy

**Keywords:** Bipolar disorder, Inflammation, Monocyte-to-lymphocyte ratio, Neutrophil-to-lymphocyte ratio, Platelet-to-lymphocyte ratio

## Abstract

**Aims:**

Bipolar disorder (BD) may be linked to immune-inflammatory dysregulation. Recently, complete blood count (CBC)-based inflammation indexes—neutrophil-to-lymphocyte ratio (NLR), monocyte-to-lymphocyte ratio (MLR), and platelet-to-lymphocyte ratio (PLR)—have emerged as potential, reproducible, and cost-effective markers for mental disorders. This study thus aimed to investigate the relationship of NLR, MLR, and PLR with manic and depressive symptom severity in people with BD, jointly testing the interactions with relevant clinical variables.

**Methods:**

We included inpatients with BD aged ≥ 18 consecutively hospitalized from May 2020 to March 2025. CBC-based ratios were calculated from fasting blood samples. Structural equation modelling (SEM) was performed to test the relationships of CBC-based ratios with manic and depressive symptom severity—assessed by the Young Mania Rating Scale (YMRS) and the Montgomery–Åsberg Depression Rating Scale (MADRS), respectively—accounting for age, sex, body mass index, alcohol/substance use disorders, and psychotropic medication doses.

**Results:**

We included 175 participants (mean age 46.8 ± 16.1 years; 48.6% males), 126 with a manic episode and 49 with a depressive episode. The MLR was higher in mania than in depression (p = 0.019), while no significant differences emerged for NLR and PLR. The SEM showed that greater YMRS scores were associated with higher NLR (coeff. = 0.077, p < 0.001) and MLR (coeff. = 0.096, p < 0.001), and lower PLR (coeff. = –0.088, p < 0.001). Moreover, higher MADRS scores were associated with lower MLR (coeff. = –0.189, p < 0.001) and higher PLR (coeff. = 0.123, p < 0.001).

**Conclusion:**

This study provides novel insights into the differential associations of NLR, MLR, and PLR with symptom severity across manic and depressive episodes, underscoring a complex immune-inflammatory dysregulation in BD. Notwithstanding generally small coefficients, our findings suggest that CBC-based ratios may represent accessible indexes to monitor mood state severity. Further investigation into their clinical utility is needed.

**Supplementary Information:**

The online version contains supplementary material available at 10.1007/s00406-025-02129-2.

## Introduction

Bipolar disorder (BD) is a chronic and severe mental disorder characterized by recurrent manic and depressive episodes [1, 2]. Although the complex pathophysiology of BD remains only partially understood, its relapsing–remitting nature seems underpinned by immune-inflammatory activation [1, 3, 4]. BD has been repeatedly associated with increased levels of pro-inflammatory cytokines, chemokines, and acute-phase proteins, along with elevated oxidative and nitrosative stress, and activation of the kynurenine pathway, particularly during acute mood episodes [5–8].

On the other hand, basic measures from routinely collected blood sample tests, such as the complete blood count (CBC), have been recently described as valuable sources of information for assessing immune-inflammatory processes [9, 10], making them practical and cost-effective instruments for both research and clinical monitoring. In particular, CBC-based ratios, including the neutrophil-to-lymphocyte ratio (NLR), the monocyte-to-lymphocyte ratio (MLR), and the platelet-to-lymphocyte ratio (PLR), have emerged as particularly informative. These ratios have shown significant correlations with markers such as C-reactive protein (CRP) [11], which has itself been linked to acute states, symptom severity, and increased risk of relapse in conditions including schizophrenia [12, 13] and BD [14, 15], thereby underscoring their potential clinical relevance. In addition, compared to absolute cell counts, CBC-based ratios are considered more stable and sensitive indicators [16, 17].

As a result, NLR, MLR, and PLR are increasingly being investigated in mental health research [18, 19], and evidence on their role in BD has accumulated [3, 20]. These markers are being studied for their potential ability to distinguish people with BD from healthy controls [21] and from those with unipolar depression [3, 22], to differentiate between manic and depressive episodes [23, 24], and to examine the interplay between inflammatory status and pharmacological treatments [25, 26]. Despite these efforts, findings have been inconsistent across studies so far [3, 26]. In particular, the key issue about the potential association between CBC-based ratios and symptom severity in BD remains unclear [27]. Moreover, although it is known that inflammatory markers correlate with many clinical variables, including age and sex [28, 29], body composition and metabolic status [29, 30], alcohol (AUD) and substance use (SUD) disorders [31, 32], and psychopharmacological agents [33–35], no study to date has evaluated how these factors may influence or moderate the relationship between inflammation and symptom severity in BD.

To address this knowledge gap, we conducted an observational study aimed to investigate the relationship of NLR, MLR, and PLR with manic and depressive symptom severity in a representative sample of inpatients with BD, also examining the potential moderating effects of key clinical variables such as age, sex, body mass index (BMI), comorbid AUD and SUD, and psychotropic treatment. Benefitting from a structural equation modelling (SEM) approach, we aimed to provide a wider understanding of the role of inflammation in BD and to identify accessible biomarkers that could inform clinical assessment and treatment planning. We hypothesized that manic symptom exacerbation would be positively associated with NLR and MLR, indicating increasing systemic and central inflammation; also, we expected worsening depressive states to correlate negatively with MLR, indicating disrupted monocyte function, and positively with PLR, reflecting monoaminergic dysregulation and endothelial dysfunction [36–39].

## Material and methods

This cross-sectional study was designed and reported following the “Strengthening the Reporting of Observational studies in Epidemiology (STROBE)” statement [40]. It was carried out in accordance with the Code of Ethics of the World Medical Association (Declaration of Helsinki) [41]. It was approved by the local Ethics Committee (“Comitato Etico Territoriale Area 3”, Milan) as a part of the broader Northern Milan Area Cohort (NOMIAC) project (registration number: 672-17,112,020) [42, 43]. Written informed consent was collected for the processing of personal data as part of routine clinical care.

### Setting and eligibility criteria

We included individuals consecutively admitted for inpatient treatment from May 2020 to March 2025 to the two acute inpatient units (27 total beds) of the Department of Mental Health and Addictions of the Nord Milano Health and Social Care Trust, which provides mental health care to people living in highly urbanized districts of the northern area of the Metropolitan City of Milan, covering a catchment area of about 280,000 inhabitants.

Subjects were included if they: (i) were ≥ 18 years old; (ii) were diagnosed with BD according to DSM-5 criteria [44]; (iii) were admitted for an acute mood episode; (iv) underwent routine blood sampling and psychometric evaluations on the first full day of hospitalization; (v) were able to provide written informed consent. We excluded people with: (i) intellectual disability; (ii) neurocognitive disorder; (iii) no CBC data available; (iv) ongoing inflammatory/infectious diseases; (v) pregnancy or breastfeeding. For study participants with multiple admissions, we used data from the first recorded hospitalization with available CBC data.

### Data collection

We collected information on age, sex (classified as male or female as assigned at birth [45]), socio-demographic and clinical features from clinical interviews, electronic medical records, and chart review. Trained assessors (part of the “NOMIAC Investigators” staff) evaluated candidate subjects for BD diagnosis, the ongoing manic or depressive episode, and the presence of DSM-5 mixed features (MFs) using the Structured Clinical Interview for DSM-5 (SCID-5) [46]. The severity of manic and depressive symptoms was assessed using the Young Mania Rating Scale (YMRS) [47] and the Montgomery-Åsberg Depression Rating Scale (MADRS) [48], respectively. Data on psychopharmacological treatment (agents and doses) were retrieved. The ratio between the prescribed daily dose (PDD) and the defined daily dose (DDD), according to the WHO ATC/DDD index, last updated on 27 December 2024 [https://atcddd.fhi.no/atc_ddd_index/, accessed 14 April 2025], was used to estimate the equivalent daily doses of antipsychotics, mood stabilizers, and antidepressants (PDD/DDD ratio).

Blood samples were routinely collected around 8.00 a.m., after an overnight fast. Haematochemical analyses employed a fluorescence flow cytometry technique performed on fully automated haematology workstation XN-3000 (Dasit) at the two units of the Clinical Chemical Analysis Laboratory of the Trust.

CBC-based ratios were estimated as follows: the NLR was calculated dividing the absolute neutrophil count (ANC) by the absolute lymphocyte count (ALC); the MLR dividing the absolute monocyte count (AMC) by the ALC; the PLR dividing the absolute platelet count (APC) by the ALC.

Anonymized data were included in a standardized extraction template and double-checked to ensure accuracy.

### Statistical analyses

Standard descriptive statistics were used to characterize the study sample. Univariate analyses were used to estimate the potential differences in relevant variables between individuals with manic and depressive episodes. Pearson’s χ^2^ and Fisher’s exact tests were used for categorical variables, while Student’s t-test or Welch’s t-test (consistently with data distribution) for continuous variables.

A latent variable SEM approach was employed to examine the interplay between CBC-based ratios and symptom severity, jointly considering the impact of different clinical domains while simultaneously accounting for measurement error. Due to the skewed distribution of CBC data, NLR, MLR, and PLR values were log-transformed before inclusion in the SEM. Latent variables for YMRS and MADRS factors were derived as a function of manifest (observed) variables. Standardized coefficients (coeff.) and p-values were estimated. First, age and sex [49] were included in the model. Then, other covariates known to influence CBC-based ratios—namely BMI [50, 51], AUD/SUD [52], and PDD/DDD ratios of antipsychotics [53], mood stabilizers [54], and antidepressants [55]—were included in the final model, concomitantly modelling the influence of pharmacotherapy [56] and AUD/SUD [57, 58] on symptom severity, as well as of psychotropic drugs on BMI [59]. In addition, we allowed for intraclass correlation, considering that observations may not be independent within subgroups by polarity (manic vs. depressive).

Statistics were performed with Stata, Release 18 [StataCorp., 2023. Stata Statistical Software: Release 18. College Station, TX: StataCorp LLC].

## Results

### Study sample and comparison between manic and depressive subgroups

Among 190 subjects with BD consecutively admitted to our inpatient units at least once during the study period for acute manic or depressive relapse, one was younger than 18 years of age at admission, six did not have data available on the CBC, and eight had an ongoing inflammatory or infectious disease. Ultimately, 175 participants met the inclusion criteria and were included in this study.

**Fig. 1:**
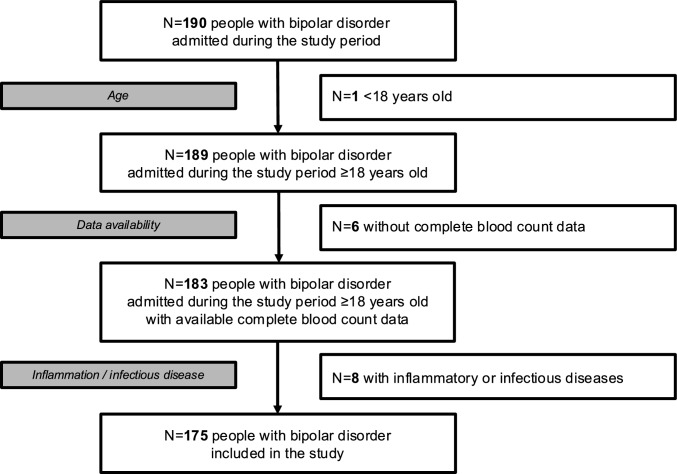
Flow chart of participant inclusion process. *N* number of subjects

The flow chart of participant inclusion process is displayed in Fig. 1.

The participants had a mean age of 46.8 years (SD = 16.1) and nearly half of them were males (48.6%). The vast majority (85.1%) were diagnosed with BD type I. The mean age at onset was 30.4 years (SD = 11.3). One hundred and twenty-six participants were admitted for a manic episode and 49 for a depressive episode. In the manic subgroup, the mean YMRS score was 29.1 (SD = 9.8), while the mean MADRS score was 9.0 (SD = 7.5); DSM-5 mixed features were present in 21 subjects (16.7%). In the depressive subgroup, subjects had a mean MADRS score of 29.3 (SD = 8.3) and a mean YMRS score of 4.7 (SD = 6.6); DSM-5 mixed features occurred in 10 individuals (20.4%). 46.9% of the depressive group had a diagnosis of BD type I (p < 0.001). SUD was more likely in the manic subgroup (p = 0.015). Notably, AMC (p = 0.020) and MLR values (p = 0.019) were higher in mania than in depression.

Overall and subgroup characteristics, as well as the univariate comparison between manic and depressive subgroups, are reported in Table 1.

**Table 1:** Socio-demographic, clinical, and biological characteristics of the whole sample and differences between participants with manic and depressive episodes

Variable		Total sample	Mania	Depression	Tests for subgroup differences
Number of subjects	*N (%)*	175 (100%)	126 (72.0%)	49 (28.0%)	–
Age	*N* *Mean* ± *SD* *Median (Q1–Q3)*	175 46.8 ± 16.1 48.4 (34.4–59.5)	126 46.4 ± 15.8 47.6 (34.7–56.4)	49 47.9 ± 16.8 48.4 (34.3–60.1)	t = 0.547 p = 0.586
Male gender	*n/N (%)*	85/175 (48.6%)	67/126 (53.2%)	18/49 (36.7%)	χ^2^ = 3.817 p = 0.051
Higher education	*n/N (%)*	33/165 (20.0%)	24/117 (20.5%)	9/48 (18.8%)	χ^2^ = 0.066 p = 0.797
Unemployed	*n/N (%)*	66/173 (38.2%	50/125 (40.0%)	16/48 (33.3%)	χ^2^ = 0.653 p = 0.419
Living alone	*n/N (%)*	58/173 (33.5%)	38/124 (30.6%)	20/49 (40.8%)	χ^2^ = 1.630 p = 0.202
In a relationship	*n/N (%)*	66/173 (38.2%)	47/126 (37.3%)	19/47 (40.4%)	χ^2^ = 0.142 p = 0.707
BD type I	*n/N (%)*	149/175 (85.1%)	126/126 (100%)	23/49 (46.9%)	**p < 0.001** ^a^
Age at onset	*N* *Mean* ± *SD* *Median (Q1–Q3)*	147 30.4 ± 11.3 29 (22–37)	112 30.4 ± 11.0 29 (22–36.5)	35 30.2 ± 12.3 27 (20–40)	t = –0.090 p = 0.929
Duration of illness	*N* *Mean* ± *SD* *Median (Q1–Q3)*	147 16.1 ± 12.9 13.5 (5–25)	112 15.6 ± 13.3 13 (3–25)	35 17.7 ± 11.8 16.5 (9.5–25)	t = 0.918 p = 0.362
Manic polarity at first episode	*n/N (%)*	87/137 (63.5%)	80/106 (75.5%)	7/31 (22.6%)	χ^2^ = 28.951 **p < 0.001**
Family history of mood disorders	*n/N (%)*	76/175 (43.4%)	53/126 (42.1%)	23/49 (46.9%)	χ^2^ = 0.341 p = 0.559
History of suicide attempts	*n/N (%)*	33/175 (18.9%)	14/126 (11.1%)	19/49 (38.8%)	χ^2^ = 17.646 **p < 0.001**
Current alcohol use disorder	*n/N (%)*	19/175 (10.9%)	16/126 (12.7%)	3/49 (6.1%)	p = 0.283^a^
Current substance use disorder	*n/N (%)*	35/175 (20.0%)	31/126 (24.6%)	4/49 (8.2%)	χ^2^ = 5.960 **p = 0.015**
YMRS total score at admission^b^	*N* *Mean* ± *SD* *Median (Q1–Q3)*	175 22.2 ± 14.2 23 (13–34)	126 29.1 ± 9.8 28 (21–37)	49 4.7 ± 6.6 2 (0–7)	t = –19.108 **p < 0.001**
MADRS total score at admission^c^	*N* *Mean* ± *SD* *Median (Q1–Q3)*	175 14.7 ± 12.0 12 (5–23)	126 9.0 ± 7.5 8 (3–14)	49 29.3 ± 8.3 30 (26–34)	t = 14.890 **p < 0.001**
WBC (× 10^9^/L)	*N* *Mean* ± *SD* *Median (Q1–Q3)*	175 8.19 ± 2.45 8.12 (6.27–9.65)	126 8.30 ± 2.50 8.21 (6.46–9.86)	49 7.90 ± 2.32 7.90 (6.27–9.29)	t = –0.891 p = 0.374
ANC (× 10^9^/L)	*N* *Mean* ± *SD* *Median (Q1–Q3)*	175 5.16 ± 2.06 4.92 (3.70–6.31)	126 5.28 ± 2.14 5.05 (3.74–6.66)	49 4.86 ± 1.82 4.70 (3.67–6.02)	t = –1.071 p = 0.286
ALC (× 10^9^/L)	*N* *Mean* ± *SD* *Median (Q1–Q3)*	175 2.16 ± 0.86 2.00 (1.55–2.65)	126 2.13 ± 0.83 1.95 (1.55–2.50)	49 2.25 ± 0.93 2.11 (1.60–2.79)	t = 0.618 p = 0.538
AMC (× 10^9^/L)	*N* *Mean* ± *SD* *Median (Q1–Q3)*	175 0.66 ± 0.21 0.63 (0.52–0.77)	126 0.68 ± 0.21 0.66 (0.55–0.80)	49 0.60 ± 0.18 0.59 (0.50–0.69)	t = –2.342 **p = 0.020**
APC (× 10^9^/L)	*N* *Mean* ± *SD* *Median (Q1–Q3)*	174 254.7 ± 69.2 246 (204–295)	126 256.8 ± 72.4 247.5 (209–296)	48 249.2 ± 60.3 238 (201.5–294)	t = –0.413 p = 0.680
NLR	*N* *Mean* ± *SD* *Median (Q1–Q3)*	175 2.77 ± 1.77 2.41 (1.52–3.40)	126 2.85 ± 1.76 2.56 (1.71–3.44)	49 2.59 ± 1.77 2.14 (1.37–3.03)	t = –1.243 p = 0.216
MLR	*N* *Mean* ± *SD* *Median (Q1–Q3)*	175 0.34 ± 0.17 0.30 (0.24–0.39)	126 0.36 ± 0.18 0.32 (0.24–0.41)	49 0.30 ± 0.12 0.28 (0.22–0.31)	t = –2.363 **p = 0.019**
PLR	*N* *Mean* ± *SD* *Median (Q1–Q3)*	174 135.1 ± 61.1 123.8 (89.2–165.0)	126 137.6 ± 60.6 128.5 (94.1–170.3)	48 125.8 ± 64.4 112.3 (86.9–144.3)	t = –0.826 p = 0.410
Antipsychotics	*n/N (%)*	105/175 (60.0%)	71/126 (56.4%)	34/49 (69.4%)	χ^2^ = 2.499 p = 0.114
Second-generation antipsychotics	*n/N (%)*	92/175 (52.6%)	61/126 (48.4%)	31/49 (63.3%)	χ^2^ = 3.121 p = 0.077
First-generation antipsychotics	*n/N (%)*	32/175 (18.3%)	27/126 (21.4%)	5/49 (10.2%)	χ^2^ = 2.975 p = 0.085
Antipsychotic PDD/DDD ratio	*N* *Mean* ± *SD* *Median (Q1–Q3)*	175 0.56 ± 0.70 0.25 (0–1)	126 0.63 ± 0.78 0.29 (0–1.00)	49 0.39 ± 0.40 0.25 (0–0.67)	t = –2.677 **p = 0.008**
Mood stabilizers	*n/N (%)*	76/175 (43.4%)	47/126 (37.3%)	29/49 (59.2%)	χ^2^ = 6.876 **p = 0.009**
Mood stabilizer PDD/DDD ratio	*N* *Mean* ± *SD* *Median (Q1–Q3)*	175 0.32 ± 0.46 0 (0–0.67)	126 0.27 ± 0.43 0 (0–0.51)	49 0.44 ± 0.52 0.25 (0–0.85)	t = 2.065 **p = 0.042**
Antidepressants	*n/N (%)*	34/175 (19.4%)	11/126 (8.7%)	23/49 (46.9%)	**p < 0.001 ** ^a^
Antidepressant PDD/DDD ratio	*N* *Mean* ± *SD* *Median (Q1–Q3)*	175 0.25 ± 0.67 0 (0–1)	126 0.10 ± 0.45 0 (0–0)	49 0.65 ± 0.94 0 (0–1)	t = 3.900 **p < 0.001**
BMI	*N* *Mean* ± *SD* *Median (Q1–Q3)*	132 26.1 ± 5.4 24.7 (22.4–29.7)	90 26.0 ± 5.3 24.6 (22.4–29.7)	42 26.4 ± 5.7 25.3 (22.5–29.6)	t = 0.445 p = 0.657
Hypertension	*n/N (%)*	30/175 (17.1%)	20/126 (15.9%)	10/49 (20.4%)	χ^2^ = 0.511 p = 0.475
Dyslipidaemia	*n/N (%)*	15/174 (8.6%)	9/125 (7.2%)	6/49 (12.2%)	χ^2^ = 1.137 p = 0.286
Diabetes	*n/N (%)*	17/175 (9.7%)	13/126 (10.3%	4/49 (8.2%)	χ^2^ = 0.187 p = 0.666
Hypothyroidism	*n/N (%)*	17/175 (9.7%)	9/126 (7.1%)	8/49 (16.3%)	χ^2^ = 3.393 p = 0.065

Tests for subgroup differences for WBC, ANC, ALC, AMC, APC, NLR, MLR, and PLR were performed with log-transformed data. t values were obtained from Student’s t-tests or Welch’s t-tests according to data distribution; χ^2^ values were obtained from χ^2^ tests. p-values < 0.05 are highlighted in bold.

*ALC* absolute lymphocyte count, *AMC* absolute monocyte count, *ANC* absolute neutrophil count, *APC* absolute platelet count, *BD* bipolar disorder, *BMI* body mass index, *MADRS* Montgomery–Åsberg Depression Rating Scale, *MLR* monocyte-to-lymphocyte ratio, *NLR* neutrophil-to-lymphocyte ratio, *PDD/DDD ratio* ratio between the prescribed daily dose (PDD) and the defined daily dose (DDD), *PLR* platelet-to-lymphocyte ratio, *WBC* White blood cell count, *YMRS* Young Mania Rating Scale, *N,n* number of subjects with data available for each variable, *Q1* first quartile, *Q3* third quartile, *SD* standard deviation.

^a^Fisher’s exact test’s p-value

^b^Manic episode with mixed features: 21/126 (16.7%)

^c^Depressive episode with mixed features: 10/49 (20.4%)

### Structural equation models

In the SEM adjusted for age and sex, higher YMRS scores were associated with higher NLR (coeff. = 0.042, p = 0.040) and MLR (coeff. = 0.180, p < 0.001) as well as with lower PLR (coeff. = –0.115, p < 0.001). Concerning depressive symptoms, participants with greater MADRS scores were more likely to show higher NLR (coeff. = 0.025, p = 0.042) and PLR (coeff. = 0.171, p < 0.001) as well as lower MLR (coeff. = –0.270, p < 0.001). Notably, higher NLR was linked to greater age (coeff. = 0.046, p < 0.001) and to male sex (coeff. = 0.208, p = 0.022) (Suppl. Table 1 in Online Resource 1).

**Fig. 2:**
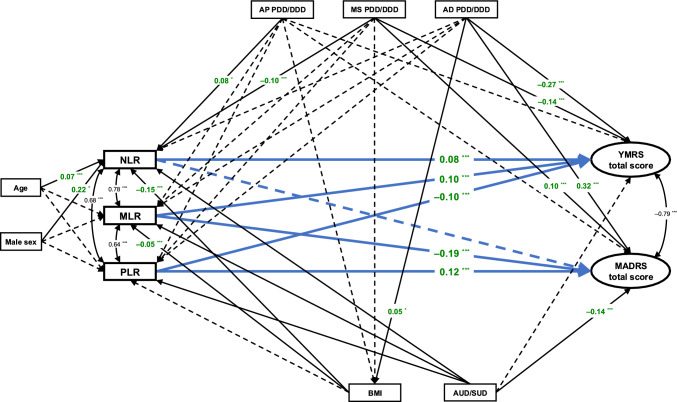
Structural equation model testing the interplay between complete blood count-based ratios, symptom severity, and relevant clinical domains. *p < 0.05; **p < 0.01; ***p < 0.001. *AD PDD/DDD* ratio between the prescribed daily dose (PDD) and the defined daily dose (DDD) of antidepressants, *AP PDD/DDD* ratio between the prescribed daily dose (PDD) and the defined daily dose (DDD) of antipsychotics, *AUD/SUD* current alcohol and/or substance use disorder, *BMI* body mass index, *MADRS* Montgomery–Åsberg Depression Rating Scale, *MLR* monocyte-to-lymphocyte ratio, *MS PDD/DDD* ratio between the prescribed daily dose (PDD) and the defined daily dose (DDD) of mood stabilizers, *NLR* neutrophil-to-lymphocyte ratio, *PLR* platelet-to-lymphocyte ratio, *YMRS* Young Mania Rating Scale

In the final SEM, greater YMRS scores were associated with higher NLR (coeff. = 0.077, p < 0.001) and MLR (coeff. = 0.096, p < 0.001), as well as with lower PLR (coeff. = –0.088, p < 0.001). Higher YMRS scores were also negatively associated with mood stabilizer (coeff. = –0.138, p = 0.004) and antidepressant (coeff. = –0.271, p = 0.004) PDD/DDD ratios. On the other hand, higher MADRS scores were associated with lower MLR (coeff. = –0.189, p < 0.001) and higher PLR (coeff. = 0.123, p < 0.001). We did not find any statistically significant association between MADRS scores and NLR (coeff. = 0.013, p = 0.625) (Suppl. Table 2 in Online Resource 2). A graphical representation of the final SEM is displayed in Fig. 2.

## Discussion

This study, using a SEM approach, explored the interplay between CBC-based ratios and symptom severity in mania and bipolar depression, simultaneously accounting for relevant clinical variables including age, sex, BMI, current AUD or SUD, and psychotropic medication doses. By incorporating both observed and latent variables into the model, SEM enables the simultaneous analysis of relevant paths, possibly exposing indirect effects on symptom severity in mania and bipolar depression. This study showed many meaningful associations between CBC-based ratios and symptom severity in people with BD: accounting for the complex interactions of both CBC-based ratios and mood symptoms with age, sex, BMI, AUD/SUD, and medications, we found that manic symptoms were positively correlated with NLR and MLR, but negatively associated with PLR, while greater depressive symptoms were associated with lower MLR and higher PLR.

The NLR reflects the interplay between innate (neutrophil-driven) and adaptive (lymphocyte-mediated) immune responses, with elevated values suggesting systemic immune-inflammatory activation [60, 61]. Despite the relative paucity, heterogeneity, and often reductive framing of studies examining the NLR during acute phases of BD, our findings seem consistent with the existing literature. Indeed, it has been shown that manic episodes may be associated with increased ANC along with reduced ALC [27]. Consistently, meta-analytic data indicate that mania is associated with higher NLR than bipolar depression (as well as euthymia) [21]. Furthermore, elevated baseline NLR seems to predict greater frequency of manic episodes but not of depressive ones in longitudinal investigations [62]. A growing body of literature indicates a pro-inflammatory state that is more severe in mania than in bipolar depression [3, 63]. Increased levels of systemic inflammation markers have been associated with manic states [3, 64, 65] and seem to correlate with higher YMRS scores but not with more severe depressive symptoms [66]. However, findings as for euthymic and depressive phases remain less clear [3, 64–66]. The specific association between NLR and manic, rather than depressive, symptoms observed in our study may support the hypothesis that systemic inflammation grows in parallel with manic symptom severity [3]. On the other hand, the positive association between MADRS scores and NLR observed in the age- and sex-adjusted SEM, but not when modelling also BMI, AUD/SUD, and medication doses, suggests that the moderating role of different clinical and treatment factors must be taken into account when exploring the relationship between systemic inflammation and depressive symptom severity in BD.

Similarly to the NLR, the MLR reflects the interplay between the non-specific inflammatory cascade and the adaptive immune response [67]. However, while the NLR is more representative of general inflammation, the MLR is more closely associated with brain-specific inflammation. Indeed, monocytes are known for their plasticity in adapting to environmental stimuli [68] and play a critical role in immune regulation, tissue repair, and the activation of microglia, infiltrating the brain and contributing to microglial priming and neurotoxic cytokine release [69, 70]. As such, the AMC is considered an indirect marker of microglia activation in the central nervous system, thus representing a peripheral correlate of neuroinflammation [71, 72]. Our simultaneous analysis of relevant paths through the SEM revealed the interplay between MLR and both manic and depressive symptom severity. However, these associations operated in opposite directions: manic symptoms were positively correlated with MLR, while depressive symptom severity was negatively associated with it. The former finding is in line with the evidence suggesting a positive correlation between heightened inflammatory markers—including IL-6 [73, 74], IL-2 [74], and soluble cytokine receptors [75, 76]—and manic severity. Conversely, the inverse association between depressive symptoms and the MLR may seem counterintuitive, as neuroinflammation has been reported in both mood poles [3, 63]. Nonetheless, this result may indicate that monocyte-related response becomes progressively disrupted relative to the adaptive immune system as depression worsens. Consistently, some studies hint at a suppression of immune signalling in bipolar depression that becomes more important as its severity increases. For instance, Wu et al. reported progressively declining levels of pro-inflammatory (such as IL-6, IL-17, and monocyte chemoattractant protein-1 (MCP-1)) and anti-inflammatory (e.g., IL-10) cytokines as the severity of bipolar depression increases, indicating a downward trajectory from mildly blunted immune activation to complete dysregulation (or “exhaustion”) [37]. Since monocytes produce both pro-inflammatory cytokines (e.g., IL-6) and anti-inflammatory mediators (as IL-10) and secrete chemokines like MCP-1 to recruit other immune cells to sites of inflammation [77, 78], these changes across bipolar depression severity suggest an escalating disruption in monocyte-mediated immune signalling. In sum, the progressive decline in MLR with increasing depressive symptoms observed in our study supports a model in which worsening bipolar depression is not driven by inflammation per se, but by a dysregulation or collapse of monocyte function instead. While this diverges from classic inflammatory models, it may indicate a distinct neural immune-inflammatory activation pattern in bipolar depression, highlighting the potential value of targeting immune balance and monocyte restoration rather than focusing solely on inflammation.

Concerning PLR, our SEM analysis showed that manic symptoms were negatively associated with PLR while depressive symptoms were positively associated with that. Beyond their role in aggregation, platelets contribute to immune responses by mediating the recruitment and activation of neutrophils and macrophages. The PLR has hence emerged as an indicator of systemic inflammation and of endothelial dysfunction (both observed in mood disorders) that could affect the trafficking of peripheral inflammatory markers to the brain [79, 80]. Platelets show an enzymatic pathway similar to dopaminergic neurons, can store and release neurotransmitters such as serotonin, glutamate, and dopamine [81], and also contain monoamine oxidase type B [82]. This may at least partly explain the positive association between PLR and depression severity, in line with the established role of monoamines in mood disorders [83]. Moreover, some studies have reported higher PLR in individuals with severe major depressive disorder compared to those with milder forms [36, 38], indicating a possible link between elevated PLR and more pronounced depressive symptomatology. Evidence of higher APC in bipolar depression relative to mania [39] may further support this notion. Though these findings should be taken at a rather speculative level, considering the difficulty of discussing them against the quite inconclusive literature available [24, 84, 85], our study highlights the potential role of PLR in both manic and depressive episodes of BD and the relevant need for further research on this matter.

By revealing distinct—and, to some extent, opposite—patterns of association between CBC-based ratios and mood symptom severity in BD, this study may suggest a future integration of such accessible markers into the clinical assessment of BD to refine symptom characterization and inform personalized management strategies. Although they do not directly allow current clinical implementation, our findings may support a multidimensional, individualized approach to BD care that incorporates routinely available immune-inflammatory profiling alongside standard psychiatric assessments and relevant clinical characteristics such as BMI, AUD or SUD, and psychopharmacotherapy. The integration of peripheral inflammatory parameters into multi-domain diagnostic models—combining biological, clinical, and pharmacological data across different levels of information—can represent a promising translational approach to identify biologically informed subtypes of BD, supporting more accurate diagnostic framing and clinical management in the future [86].

While the positive associations of NLR and MLR with manic symptom severity may suggest the clinical utility of identifying and targeting systemic inflammation at the early stages, the findings concerning depressive symptoms may reflect an immune dysregulation pattern that differs from classical inflammation, calling for novel therapeutic strategies aimed to restore monocyte function and immune balance in bipolar depression. In this context, recent efforts exploring the role of anti-inflammatory and immune-modulating treatments in BD may remain a promising research direction [87]. Albeit mixed and not uniformly positive, findings from clinical trials suggest that anti-inflammatory drugs like acetylsalicylic acid and celecoxib can be effectively repurposed as adjunctive treatments in both mania and bipolar depression [87, 88], showing greater symptom improvement compared to placebo while also potentially contributing to reductions in inflammatory parameters such as serum CRP [89]. CBC-based ratios might also hold promise as predictors of treatment response in BD. Nonetheless, current evidence in this regard is still limited and preliminary [55, 90], warranting further longitudinal studies to clarify their prognostic value.

### Limitations

Some limitations must be considered. First, although the SEM approach allowed for an in-depth investigation of the complex interrelationships between clinical and biological domains, the use of cross-sectional data limits temporal or causal inferences and warrants cautious interpretation of the SEM [91]. Second, the associations, while statistically significant, had small effect sizes, suggesting that findings should be interpreted carefully. Third, our sample included only acute, hospitalized subjects, thereby excluding individuals with less severe symptomatology who are typically managed in outpatient settings and thus not capturing the full spectrum of severity, possibly limiting generalizability. Moreover, owing to the relatively limited sample size, we could not fit more potentially relevant variables, such as specific pharmacological agents or comorbid medical conditions, into the model. Finally, we did not concurrently assess a broader panel of inflammatory biomarkers, which might have further improved the understanding of the neuroimmune underpinnings of manic and depressive symptomatology in BD.

## Conclusion

This study provides novel insights into the relationship of NLR, MLR, and PLR with symptom severity across manic and depressive episodes in BD, highlighting the complex interplay between inflammation and mood symptoms in the disorder. Distinct immune-inflammatory patterns emerged, suggesting state-specific alterations that may reflect divergent mechanisms underlying mood polarity and severity. This study highlights the potential of the accessible and cost-effective CBC-based ratios as indicators of symptom severity in mania and bipolar depression. Future investigations should be aimed to clarify the temporal and causal dynamics of immune-inflammatory activation across mood phases and their potential clinical utility in tailoring interventions for BD.

## Supplementary Information

Below is the link to the electronic supplementary material.

Supplementary file1 (PDF 107 KB)

Supplementary file2 (PDF 128 KB)

## Declarations

### Data availability statement

The data that support the findings of this study are available from the corresponding author upon reasonable request.
