# Supplementary material for: Complete blood count-based inflammation indexes and symptom severity in people with bipolar disorder: an analysis based on structural equation modelling
Source: Eur Arch Psychiatry Clin Neurosci. 2025 Nov 4;276(2):561–72. doi: 10.1007/s00406-025-02129-2 (PMC12953282; doi:10.1007/s00406-025-02129-2)
Supplement: Supplementary file 1 — Supplementary file1 (PDF 107 KB) [file 406_2025_2129_MOESM1_ESM.pdf]

Suppl. Tab. 1 Structural equation model including age and sex

Outcome	Features	Standardized coefficient	95% Confidence Interval	Standard Error	z	p–value
logNLR	Age	0.046	0.026 to 0.067	0.011	4.39	**<0.001**
	Male sex	0.208	0.030 to 0.386	0.091	2.29	**0.022**
logMLR	Age	0.001	–0.160 to 0.161	0.082	0.01	0.994
	Male sex	0.206	–0.049 to 0.462	0.130	1.58	0.114
logPLR	Age	0.024	–0.083 to 0.132	0.055	0.44	0.658
	Male sex	–0.021	–0.198 to 0.157	0.091	–0.23	0.820
YMRS	logNLR	0.042	0.002 to 0.082	0.020	2.05	**0.040**
	logMLR	0.180	0.110 to 0.250	0.036	5.05	**<0.001**
	logPLR	–0.115	–0.126 to –0.103	0.006	–19.63	**<0.001**
MADRS	logNLR	0.025	0.001 to 0.048	0.012	2.03	**0.042**
	logMLR	–0.270	–0.390 to –0.150	0.061	–4.40	**<0.001**
	logPLR	0.171	0.159 to 0.182	0.006	29.67	**<0.001**
Covariance logNLR – logMLR		0.774	0.696 to 0.851	0.040	19.58	**<0.001**
Covariance logNLR – logPLR		0.674	0.607 to 0.741	0.034	19.63	**<0.001**
Covariance logMLR – logPLR		0.637	0.607 to 0.667	0.015	41.72	**<0.001**
Covariance YMRS total score – MADRS total score		–0.802	–1.115 to -0.490	0.159	–5.03	**<0.001**

BMI: body mass index; MADRS: Montgomery–Åsberg Depression Rating Scale; MLR: monocyte-to-lymphocyte ratio; NLR: neutrophil-to-lymphocyte ratio; PDD/DDD ratio: ratio between the prescribed daily dose (PDD) and the defined daily dose (DDD); PLR: platelet-to-lymphocyte ratio; YMRS: Young Mania Rating Scale.
