# Supplementary material for: Complete blood count-based inflammation indexes and symptom severity in people with bipolar disorder: an analysis based on structural equation modelling
Source: Eur Arch Psychiatry Clin Neurosci. 2025 Nov 4;276(2):561–72. doi: 10.1007/s00406-025-02129-2 (PMC12953282; doi:10.1007/s00406-025-02129-2)
Supplement: Supplementary file 2 — Supplementary file2 (PDF 128 KB) [file 406_2025_2129_MOESM2_ESM.pdf]

Suppl. Tab. 2 Structural equation model including all relevant covariates

Outcome	Features	Standardized coefficient	95% Confidence Interval	Standard Error	z	p–value
logNLR	Age	0.068	0.044 to 0.092	0.012	5.52	**<0.001**
	Male sex	0.225	0.032 to 0.417	0.098	2.29	**0.022**
	BMI	–0.150	–0.179 to –0.121	0.015	–10.19	**<0.001**
	Alcohol/substance use disorder	–0.027	–0.108 to 0.053	0.041	–0.66	0.507
	Antipsychotic PDD/DDD ratio	0.082	0.002 to 0.162	0.041	2.02	**0.044**
	Mood stabilizer PDD/DDD ratio	–0.098	–0.109 to –0.088	0.005	–18.29	**<0.001**
	Antidepressant PDD/DDD ratio	0.034	–0.266 to 0.333	0.153	0.22	0.825
logMLR	Age	0.029	–0.116 to 0.173	0.074	0.39	0.699
	Male sex	0.199	–0.073 to 0.472	0.139	1.43	0.152
	BMI	–0.053	–0.081 to –0.026	0.014	–3.85	**<0.001**
	Alcohol/substance use disorder	0.012	–0.044 to 0.068	0.029	0.42	0.677
	Antipsychotic PDD/DDD ratio	0.013	–0.160 to 0.186	0.088	0.15	0.883
	Mood stabilizer PDD/DDD ratio	–0.119	–0.311 to 0.072	0.098	–1.22	0.222
	Antidepressant PDD/DDD ratio	–0.032	–0.273 to 0.209	0.123	–0.26	0.794
logPLR	Age	0.009	–0.154 to 0.171	0.083	0.10	0.918
	Male sex	0.007	–0.145 to 0.160	0.078	0.09	0.925
	BMI	–0.051	–0.105 to 0.002	0.028	–1.86	0.063
	Alcohol/substance use disorder	–0.087	–0.253 to 0.079	0.085	–1.03	0.305
	Antipsychotic PDD/DDD ratio	–0.004	–0.132 to 0.123	0.065	–0.07	0.946
	Mood stabilizer PDD/DDD ratio	–0.043	–0.230 to 0.145	0.096	–0.44	0.657
	Antidepressant PDD/DDD ratio	–0.014	–0.191 to 0.163	0.090	–0.15	0.879
BMI	Antipsychotic PDD/DDD ratio	0.030	–0.035 to 0.094	0.033	0.9	0.370
	Mood stabilizer PDD/DDD ratio	0.011	–0.020 to 0.042	0.016	0.7	0.483
	Antidepressant PDD/DDD ratio	0.049	0.008 to 0.091	0.021	2.33	**0.020**
YMRS	logNLR	0.077	0.048 to 0.106	0.015	5.23	**<0.001**
	logMLR	0.096	0.093 to 0.099	0.001	70.05	**<0.001**
	logPLR	–0.093	–0.109 to –0.082	0.003	–33.92	**<0.001**
	Alcohol/substance use disorder	0.073	–0.109 to 0.256	0.093	0.79	0.431
	Antipsychotic PDD/DDD ratio	–0.006	–0.337 to 0.324	0.169	–0.04	0.970
	Mood stabilizer PDD/DDD ratio	–0.138	–0.230 to –0.045	0.047	–2.92	**0.004**
	Antidepressant PDD/DDD ratio	–0.271	–0.457 to –0.086	0.095	–2.87	**0.004**
MADRS	logNLR	0.013	–0.039 to 0.065	0.026	0.49	0.625
	logMLR	–0.189	–0.283 to –0.095	0.048	–3.94	**<0.001**
	logPLR	0.123	0.113 to 0.134	0.005	23.72	**<0.001**
	Alcohol/substance use disorder	–0.137	–0.212 to –0.062	0.038	–3.58	**<0.001**
	Antipsychotic PDD/DDD ratio	–0.120	–0.312 to 0.073	0.098	–1.22	0.223
	Mood stabilizer PDD/DDD ratio	0.103	0.090 to 0.117	0.007	14.99	**<0.001**
	Antidepressant PDD/DDD ratio	0.323	0.308 to 0.337	0.007	44.46	**<0.001**
Covariance logNLR – logMLR		0.777	0.697 to 0.857	0.041	19.06	**<0.001**
Covariance logNLR – logPLR		0.679	0.624 to 0.734	0.028	24.21	**<0.001**
Covariance logMLR – logPLR		0.639	0.598 to 0.681	0.021	30.19	**<0.001**
Covariance YMRS total score – MADRS total score		–0.788	–1.072 to 0.505	0.145	–5.45	**<0.001**

BMI: body mass index; MADRS: Montgomery–Åsberg Depression Rating Scale; MLR: monocyte-to-lymphocyte ratio; NLR: neutrophil-to-lymphocyte ratio; PDD/DDD ratio: ratio between the prescribed daily dose (PDD) and the defined daily dose (DDD); PLR: platelet-to-lymphocyte ratio; YMRS: Young Mania Rating Scale.
